# Dissociable Effects of Endogenous and Exogenous Attention on Crowding: Evidence from Event-Related Potentials

**DOI:** 10.3390/brainsci14100956

**Published:** 2024-09-24

**Authors:** Mingliang Gong, Tingyu Liu, Yingbing Chen, Yingying Sun

**Affiliations:** School of Psychology, Jiangxi Normal University, Nanchang 330022, China; 202040100260@jxnu.edu.cn (T.L.); 202243200004@jxnu.edu.cn (Y.C.); 202343200011@jxnu.edu.cn (Y.S.)

**Keywords:** visual crowding, endogenous attention, exogenous attention, cue validity, ERP

## Abstract

Background/Objectives: Crowding is a common visual phenomenon that can significantly impair the recognition of objects in peripheral vision. Two recent behavioral studies have revealed that both exogenous and endogenous attention can alleviate crowding, but exogenous attention seems to be more effective. Methods: The present study employed the event-related potential (ERP) technique to explore the electrophysiological characteristics of the influence of these two types of attention on crowding. In the experiment, participants were required to judge whether the letter “T” was upright or inverted, which may be preceded by an exogenous cue or an endogenous cue indicating the location of the target letter. Results: The behavioral results showed that while exogenous cues reduced crowding in all stimulus onset asynchronies (SOAs), endogenous attention took effects only in long SOA. The ERP results indicated that both endogenous and exogenous cues significantly alleviated the inhibition of visual crowding on the N1 component. However, the endogenous cue was effective only under long SOA, while the exogenous cue was effective only under short SOA conditions. In addition, invalid exogenous cues induced a larger P3 wave amplitude than valid ones in the short SOA condition, but endogenous attention did not show such a difference. Conclusions: These results indicate that both endogenous and exogenous attention can alleviate the effects of visual crowding, but they differ in effect size and temporal dynamics.

## 1. Introduction

You may have encountered situations where finding a friend in an open space is relatively straightforward, but the task becomes increasingly challenging when the friend is amidst a dense crowd. This challenge is attributed to a phenomenon called visual crowding, which is characterized by the detrimental influence of neighboring stimuli on the ability to visually discriminate a target stimulus. Crowding is ubiquitous and can occur among various types of visual stimuli [1]. It significantly hampers our capacity to discriminate among stimuli and has been regarded as a fundamental constraint in the recognition of objects in the peripheral visual field [1,2]. As such, a significant body of research has focused on identifying factors that influence the degree of crowding, aiming to mitigate its impact on our daily activities. These factors encompass the nature of task [3,4,5], the features of visual stimuli [4,6,7,8], and the allocation of attention [9,10,11,12,13]. Particularly, attention has emerged as a pivotal factor in this context, exerting a crucial influence on the perception of objects in crowded visual scenes.

Attention can be categorized into two primary types: endogenous attention and exogenous attention [14]. Endogenous attention is motivated by internal factors such as goals or expectations that necessitate a top-down, active allocation of attention [15]. Conversely, exogenous attention is driven by salient external cues, triggering a bottom-up, reflective orienting of attention. Given that it functions automatically and may be used with reflex-like circuitry to respond rapidly to behaviorally relevant stimuli, it is considered to have a phylogenetically ancient capacity [16]. According to the characteristics of these two types of attention, Posner [14] developed the well-known Posner cueing task. In the task, participants are required to react as fast as possible to a peripheral target that is preceded by either a central cue (eliciting endogenous attention) or peripheral cue (eliciting exogenous attention). The central cue typically involves an arrow presented at fixation, which, in most trials, accurately indicates the target location. Since orienting relies on the symbolic significance and predictive nature of the arrow, it emerges internally and is considered voluntary and subject to control. On the other hand, the peripheral cue typically manifests as a brightening or a point that appears at the location of the target. Despite the absence of a direct task-driven goal to shift attention towards this point, it can trigger reflexive and automatic orienting in a bottom-up manner [17].

Moreover, exogenous attention demonstrates a much faster cueing effect than endogenous attention. Specifically, exogenous attention typically reaches its peak effect around 120 ms after cue onset [16,18,19], whereas endogenous attention takes approximately 300 ms to manifest after cue onset and can persist for an extended duration depending on one’s need [16,19,20]. This suggests that exogenous and endogenous attention possess distinct temporal characteristics and underlying mechanisms (for a review, see [21]). Both types of attention, endogenous and exogenous, have been demonstrated to modulate visual crowding, albeit with a preponderance of existing studies focusing on the effect of exogenous attention. When attention is directed to a particular region by a peripheral cue, visual crowding within that region reduces [11,12,13,22,23,24,25]. Endogenous attention can also reduce the crowding effect, but this conclusion has been directly supported by only one study [26].

Recently, two separate studies have directly compared the effects of exogenous and endogenous attention on crowding [9,27]. Gong et al.’s [9] study integrated a Posner cueing task with a crowding task, varying SOA between cue and target to explore the differential effects of exogenous and endogenous attention on crowding. Their results revealed that both attentional cues significantly improved accuracy in discriminating the orientation of the letter “T”, with exogenous attention demonstrating a higher enhancement than endogenous attention. On the other hand, Bowen et al. [27] employed an anticueing task in which participants had to discriminate the orientation of a Gabor patch. They found that while both types of attention reduced response time (RT), only exogenous attention decreased the critical spacing of crowding. Taken together, these two studies demonstrate that both endogenous and exogenous attention can alleviate crowding, while exogenous attention seems to show some advantages. It is noteworthy that the two studies employed distinct tasks (discrimination of the orientation of the letter “T” versus Gabor patches) and utilized different behavioral measures (accuracy versus RT and critical spacing).

However, the studies conducted by Brown et al. [27] and Gong et al. [9] focused on behavioral performance. The underlying electrophysiological differences, as well as the specific stage at which the two types of attention diverge during processing, remain uncertain. To address this question, the present study employed a paradigm similar to that used by Gong et al. [9] but utilized the ERP technique in an attempt to investigate the temporal dynamics of how the two types of attention differentially impact crowding. The SOA between the cue and the target randomly varied over a short interval (100–200 ms) or a long (300–700 ms) interval. We hypothesize that both endogenous and exogenous cues can significantly mitigate visual crowding, with exogenous attention exerting a more pronounced and rapid effect. At the electrocortical level, we focus on the components of N1 and P3. Previous research has consistently demonstrated that visual crowding can significantly suppress the amplitude of the N1 component [28,29,30]. Indeed, the decrease in N1 amplitude can be regarded as an ERP marker for the occurrence of crowding. N1 is sensitive to attention in a crowding task, as evidenced by the fact that attending to a crowded target has been shown to elicit a larger N1 amplitude [28,29]. We therefore hypothesize that both valid exogenous and valid endogenous cues attenuate the suppressive effect of crowding on the N1 component compared to invalid cues. However, the exogenous cue demonstrates this effect at both short and long SOAs, whereas the endogenous cue only shows this effect at the long SOA. For the P3 component, its amplitude has been widely used as an indicator to evaluate the amount of cognitive resources that are involved in tasks [31,32,33]. Generally, a higher amplitude of P3 is considered indicative of greater cognitive resource consumption. Directing attention towards a target, regardless of whether it is exogenous or endogenous, can alleviate visual crowding, thereby facilitating the recognition of target stimuli (see [9]). Therefore, we believe that P3 would be affected by both exogenous and endogenous attention. Specifically, we anticipate that the amplitude of the P3 wave elicited under the invalid condition will be significantly larger than that observed in the valid condition for both exogenous and endogenous cues.

## 2. Method

### 2.1. Participants

Forty-one undergraduate students were recruited, but four were excluded from the analysis due to excessive noise in their electroencephalogram (EEG) data. The remaining 37 participants (28 females; mean age = 19.73 years) were included in the analysis. All participants had normal or corrected-to-normal vision and were right-handed. None of them knew the purpose of the experiment and had never taken part in similar experiments. The study was approved by the Ethics Committee of the Institute, and the written informed consent of the participants was obtained before the experiment. Furthermore, all participants received financial compensation after the experiment.

### 2.2. Stimuli

The same letters used in Gong et al.’s study [9] were used in this study. Specifically, the capital letter “T” (0.9° × 0.9°) was used as the target stimulus, and the capital letter “H” (0.9° × 0.9°) was used as the flanking stimulus. The letters were in Sloan font and were all black. Similar to Scolari et al.’s study [11], the target letters were presented either upright or inverted. The flanker letters were presented either upright or tilted 90°, with one letter positioned above the target and one below it. The center-to-center distance between target and flanker stimuli was 1.5°. A black cross measuring 0.3° × 0.3° was used as the fixation. The exogenous attention cue was a black dot with a diameter of 0.35° in visual angle, while the endogenous attention cue was a black arrow with a length of 1°. The masking stimuli consisted of three black grids with a size of 0.9° × 0.9°.

### 2.3. Design and Procedure

The experiment adopted a 2 (cue type: exogenous attention cues vs. endogenous attention cues) × 2 (cue validity: valid vs. invalid) × 2 (SOA: short vs. long) within-subject design, with accuracy and response time serving as dependent variables. In this experiment, the cue validity was 75%, indicating that the cue and the target appeared on the same side of the fixation 75% of the time, and the target appeared on the opposite side 25% of the time. Considering that exogenous cues typically reach their peak effects about 120 ms after the onset of the cues and that endogenous attention requires at least 300 ms for deployment (e.g., [34,35]), two SOAs—a short SOA (100–200 ms) and a long SOA (300–700 ms)—were employed. In the short SOA (100–200 ms) condition, EEG signals elicited by crowded target stimuli could potentially become contaminated by EEG responses elicited by the cues. In order to better distinguish the amplitude elicited by crowded targets, non-target trials were added to obtain the cue-elicited EEG signal. In these trials, after the cue appeared, the target stimulus was not presented, and the participants did not need to make any response.

**Figure 1:**
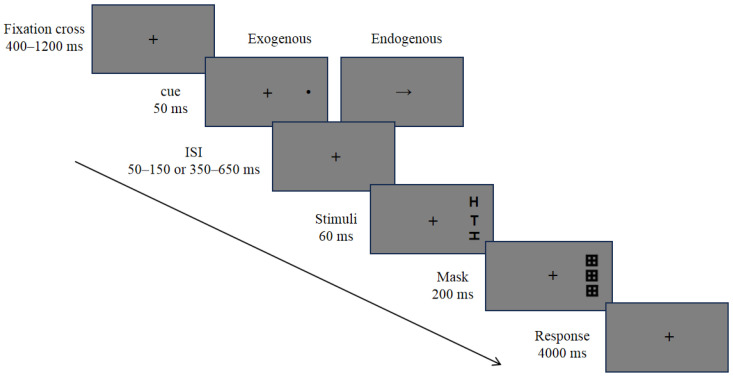
Schematic illustration of a trial in the experiment.

This experiment combined the Posner cue–target task with the visual crowding task (see Figure 1). Participants first completed a 20-trial practice session, and subsequently, they entered the formal experiment only if their accuracy in the practice session reached 75% or above. Each trial began with a fixation cross, which was presented in the center of the screen for 400–1200 ms. Subsequently, a 50 ms cue was presented. The exogenous cue was a black dot appearing 8° away from the fixation, while the endogenous cue was an arrow appearing in the center of the screen. After either a short (with ISI varying randomly between 50 and 150 ms) or long (with ISI varying randomly between 350 and 650 ms) interstimulus interval (ISI), the target and flankers appeared. They were presented for 60 ms, with three letters appearing and the target letter positioned in the middle. Next, three grid-masking stimuli were presented for 200 ms. Finally, a response screen appeared, and the participants were explicitly required to judge whether the target letter was upright or inverted as accurately as possible. Response time was not required, but they were asked to respond within a period of 4000 ms. After the reaction, a 500 ms blank screen appeared before the start of the next trial.

The whole experiment consisted of a total of 960 trials, separated into two blocks of 480 trials. Each block consisted of 360 target trials and 120 non-target trials. Short breaks were allowed after each of the 240 trials. The experiment was programmed using E-Prime 3.0.

### 2.4. Electrophysiological Recording and Analysis

EEG data were recorded using 64-conductor Ag/AgCl electrodes (FP1, FP2, AF7, AF3, AFz, AF4, AF8, F7, F5, F3, F1, Fz, F2, F4, F6, F8, FT9, FT7, FC5, FC3, FC1, FCz, FC2, FC4, FC6, FT8, FT10, T7, C5, C3, C1, Cz, C2, C4, C6, T8, TP9, TP7, CP5, CP3, CP1, CPz, CP2, CP4, CP6, TP8, TP10, P7, P5, P3, P1, Pz, P2, P4, P6, P8, PO7, PO3, POz, PO4, PO8, O1, Oz, and O2) mounted on an elastic cap manufactured by Brain Products GmbH (Gilching, Germany), with the electrodes arranged according to the International 10–20 system. Two bipolar electrode pairs were used to record vertical and horizontal electrooculography (EOG) for the detection of eye movements and blinks. Electrode impedances were below 5 KΩ during the task, and the sampling rate was 500 Hz.

EEGLAB v2020.0 [36] and Letswave 7 (www.letswave.org, accessed on 1 February 2023) were used for data processing and analysis. First, all electrodes were re-referenced offline to the average of the left and right mastoids (TP9, TP10). Second, the EEG data were filtered with a bandpass of 0.1 to 30 Hz. Independent component analysis (ICA) with the runica algorithm was used to remove eye movements and blink artifacts. Moreover, the ICA with the PCA option was performed to match the data rank to handle the rank deficiency issue [37]. For bad channels (FT9 and FT10), we first removed the two channels; then, after running ICA, the missing electrodes were interpolated. Next, the data were segmented, beginning at 200 ms before cue onset and lasting until 800 ms after cue onset. All epochs were baseline-corrected with respect to the mean voltage over the 200 ms pre-stimulus period. EEG data exceeding ±100 μV were excluded from further analysis. Finally, trial averages were calculated for each participant and condition to obtain the averaged data.

Based on the existing literature regarding electrode distributions for the N1 and P3 components (e.g., [28,31]) and considering the findings of our current study, we selected the electrodes and time windows that most accurately capture the activity characteristics of N1 and P3. Specifically, since the N1 effects of crowding are primarily observed in the occipital lobe [30] and adjacent regions in the parietal and temporal lobes [28], we opted for the same electrodes over the parietal and occipital areas identified by Ronconi et al., which include TP7, CP5, CP3, CP1, CPz, CP2, CP4, CP6, TP8, P7, P5, P3, P1, Pz, P2, P4, P6, P8, PO7, PO3, POz, PO4, PO8, O1, Oz, and O2. Given that, under crowding, N1 peaks around 200 ms post-stimulus [30] and in light of our experimental results, we selected a time window of 176–202 ms, consistent with Chicherov et al. Previous studies have primarily focused on the initial detectable effects of crowding, often overlooking the later processing stages of the P3 component. To address this, we referred to previous research on P3 (e.g., [31]) while incorporating our study’s findings, ultimately selecting a time window of 300–450 ms and the electrodes CP1, CP2, CP3, CP4, CPz, P1, P2, P3, P4, and Pz.

Additionally, to eliminate the potential contamination of EEG signals elicited by the cues on the target, the amplitude elicited by non-target trials was subtracted from the amplitude elicited by target trials in the valid condition during short-term SOA, following the subtraction procedure of Tian et al. [38]. The same operation was performed for the invalid condition.

## 3. Results

### 3.1. Behavioral Results

**Figure 2:**
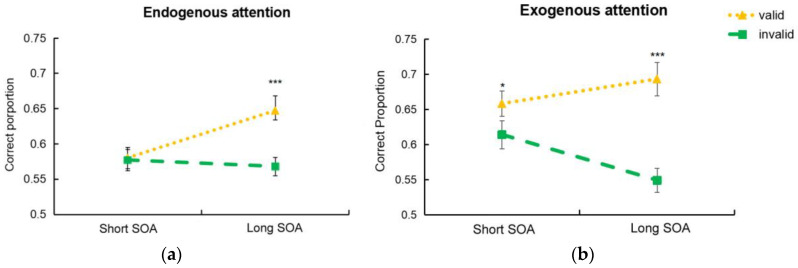
Accuracy of cue validity in endogenous (**a**) and exogenous cue (**b**) conditions. Note. *** *p* < 0.01, * *p* < 0.05.

A 2 (cue type) × 2 (cue validity) × 2 (SOA) repeated measures ANOVA was conducted, with participants’ accuracy as the dependent variable (see Figure 2). The results revealed a significant interaction between cue type and SOA [*F* (1,36) = 8.58, *p* = 0.006, $\eta_{p}^{2}$ = 0.19]. In the endogenous cue condition, participants’ accuracy for the long SOA was significantly higher than that for the short SOA (*p* = 0.014). However, in the exogenous cue condition, there was no significant difference in accuracy between the two different SOAs (*p* = 0.278). Also, the interaction between the two types of attention and cue validity was significant [*F* (1,36) = 16.05, *p* < 0.001, $\eta_{p}^{2}$ = 0.31]. Both endogenous and exogenous attention showed significantly higher accuracy in the valid cue condition than in the invalid cue condition (*p* = 0.001 and *p* < 0.001, respectively). The interaction between SOA and cue validity was also significant [*F* (1,36) = 18.47, *p* < 0.001, $\eta_{p}^{2}$ = 0.31]. Valid cues exhibited significantly higher accuracy than invalid cues in the long-SOA condition (*p* < 0.001), whereas the cue validity condition of the short SOA did not show a significant difference (*p* = 0.073). Main effects were found to be significant for cue type [*F* (1,36) = 11.44, *p* = 0.002, $\eta_{p}^{2}$ = 0.24] and cue validity [*F* (1,36) = 36.23, *p* < 0.001, $\eta_{p}^{2}$ = 0.50], but the main effect was not significant for SOA [*F* (1,36) = 0.52, *p* = 0.47, $\eta_{p}^{2}$ = 0.01].

The three-way interaction was not significant [*F* (1,36) = 0.65, *p* = 0.43, $\eta_{p}^{2}$ = 0.02]. Given that the primary focus of the present study was the differential effects of exogenous and endogenous attentional cues on visual crowding under different SOAs, additional analyses were performed. It was found that the interaction between SOA and cue validity was significant in the endogenous attention condition [*F* (1,36) = 8.87, *p* = 0.005, $\eta_{p}^{2}$ = 0.20]. The accuracy of valid cues was significantly higher than that of invalid cues in the long-duration condition (*p* < 0.001), whereas there was no significant difference between valid and invalid cues in the short-duration condition (*p* = 0.86). The interaction between SOA and cue validity was also significant in the exogenous attention condition [*F* (1,36) = 15.72, *p* < 0.001, $\eta_{p}^{2}$ = 0.30]. In both the long- and short-SOA conditions, the accuracy of valid cues was significantly higher than that of invalid cues (*p* < 0.001 and *p* = 0.015, respectively).

Since we were also interested in comparing the effectiveness of the endogenous and exogenous cues on alleviating crowding, a planned comparison was conducted. The results showed that the exogenous cue had a significantly greater impact on reducing crowding compared to the endogenous cue in both short- and long-SOA conditions (*t* (36) = 5.41, *p* < 0.001, and *t* (36) = 3.06, *p* = 0.004).

The reaction times (RTs) in different conditions were also computed (see Table 1). Then, a similar ANOVA, with RT as the dependent variable, was also carried out. The results showed that the three-way interaction was not significant [*F* (1,36) = 0.009, *p* = 0.92, $\eta_{p}^{2}$ < 0.001]. The interaction between cue type and cue validity was significant [*F* (1,36) = 7.39, *p* = 0.01, $\eta_{p}^{2}$ = 0.17]. The RTs for valid cues were significantly shorter than those for the invalid cues in both endogenous and exogenous cue conditions (*ps* < 0.001). Neither the interaction between cue type and SOA nor the interaction between SOA and cue validity were statistically significant [*F* (1,36) = 0.94, *p* = 0.34, $\eta_{p}^{2}$ = 0.025 and *F* (1,36) = 0.06, *p* = 0.81, $\eta_{p}^{2}$ = 0.002, respectively]. Meanwhile, there was a significant main effect of cue validity [*F* (1,36) = 46.92, *p* < 0.001, $\eta_{p}^{2}$ = 0.57], but neither the main effect of cue type nor that of SOA were significant [*F* (1,36) = 0.58, *p* = 0.45, $\eta_{p}^{2}$ = 0.016, and *F* (1,36) = 1.49, *p* = 0.23, $\eta_{p}^{2}$ = 0.04, respectively].

### 3.2. ERP Results

**Figure 3:**
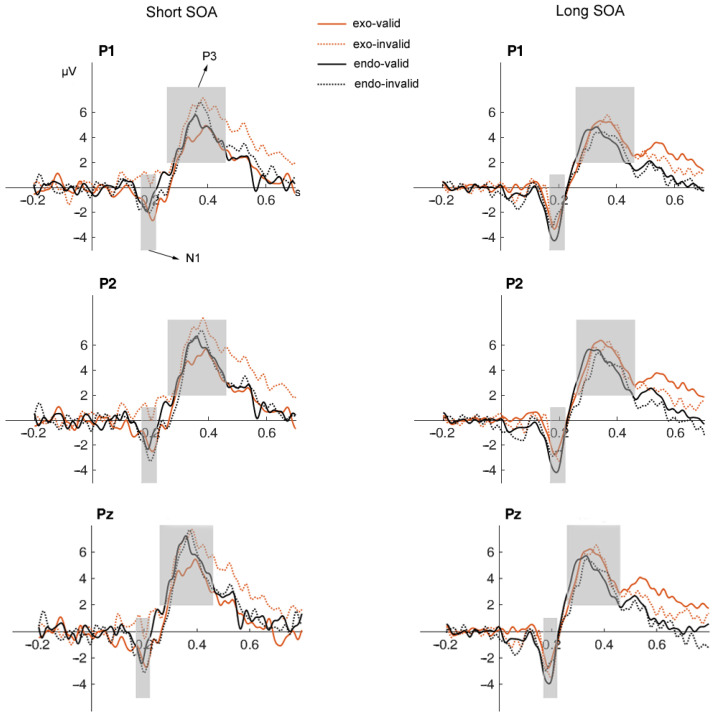
The ERP waveforms elicited by endogenous and exogenous cues at some electrode sites under different conditions of SOA. Note. The left and right columns depict conditions under short and long SOAs, respectively. Exo-valid represents a valid exogenous cue, exo-invalid represents invalid an exogenous cue, endo-valid represents a valid endogenous cue, and endo-invalid represents an invalid endogenous cue.

Some of the electrode point-averaged wave amplitudes from the experimental results are shown in Figure 3. A repeated measures ANOVA, consisting of a 2 (cue type: endogenous vs. exogenous) × 2 (cue validity: valid vs. invalid) × 2 (SOA: short vs. long) design, was conducted, with N1 and P3 amplitudes serving as the dependent variables.

**N1**. The thee-way interaction was significant [*F* (1,28) = 8.22, *p* = 0.008,${\;\eta }_{p}^{2}$ = 0.23]. Subsequent analysis revealed nonsignificant interaction between SOA and cue validity in the endogenous attention condition [*F* (1,28) = 3.47, *p* = 0.073, $\eta_{p}^{2}$ = 0.11] and in the exogenous attention condition [*F* (1,28) = 3.11, *p* = 0.089, $\eta_{p}^{2}$ = 0.10]. To examine the hypotheses of the present study, further analyses were conducted. The results revealed that there was no significant difference between the N1 amplitudes of the invalid endogenous cue and valid endogenous cue conditions in the short-SOA condition (*p* = 0.57), whereas in the long-SOA condition, the amplitude of the invalid endogenous cue was significantly smaller than that of the valid endogenous cue (*p* = 0.03). The amplitude of the invalid exogenous cue was significantly smaller than that of the valid exogenous cue in the short-SOA condition (*p* = 0.041), whereas there was no difference in N1 amplitude between the invalid exogenous cue and the valid exogenous cue in the long-SOA condition (*p* = 0.561).

In addition, significance emerged for the interaction of cue type with SOA [*F* (1,28) = 1.39, *p* = 0.248, $\eta_{p}^{2}$ = 0.05] and the interaction of cue type with cue validity [*F* (1,28) = 0.10, *p* = 0.303, η^2^ = 0.04]. In contrast, the interaction between SOA and cue validity was not significant [*F* (1,28) = 0.002, *p* = 0.968, $\eta_{p}^{2}$ < 0.001]. The main effect of SOA was significant [*F* (1,28) = 19.78, *p* < 0.001, $\eta_{p}^{2}$ = 0.41], while the main effects of cue type and cue validity were not significant [*F* (1,28) = 3.66, *p* = 0.066, $\eta_{p}^{2}$ = 0.12; *F* (1,28) = 3.39, *p* = 0.076, $\eta_{p}^{2}$ = 0.11].

**P3**. The three-way interaction was significant [*F* (1,28) = 4.95, *p* = 0.034, $\eta_{p}^{2}$ = 0.15]. Our post hoc analyses found no significant interaction between SOA and cue validity in the endogenous attention condition [*F* (1,28) = 0.01, *p* = 0.923, $\eta_{p}^{2}$ < 0.001]. Again, to further explore the hypotheses of the present study, further analyses were conducted. For both short and long SOAs, the difference in the P3 amplitude between valid and invalid cues was not statistically significant (*p* = 0.986 and *p* = 0.870, respectively). However, the interaction between SOA and cue validity was significant in the exogenous attention condition [*F* (1,28) = 7.56, *p* = 0.01, $\eta_{p}^{2}$ = 0.21]. Specifically, the P3 amplitude in the invalid cue condition was significantly higher than that in the valid cue condition in the short-SOA condition (*p* = 0.001), whereas there was no significant difference between the two in the long-SOA condition (*p* = 0.831).

The two-way interaction between SOA and cue validity was significant [*F* (1,28) = 4.27, *p* = 0.048, $\eta_{p}^{2}$ = 0.13], but the interactions between cue type and SOA [*F* (1,28) = 1.85, *p* = 0.185, $\eta_{p}^{2}$ = 0.06] and between cue type and cue validity [*F* (1,28) = 2.17, *p* = 0.15, $\eta_{p}^{2}$ = 0.07] were not significant. Moreover, the main effect of SOA was significant [*F* (1,28) = 4.35, *p* = 0.046, $\eta_{p}^{2}$ = 0.13], while the main effects of cue type and cue validity were not significant [*F* (1,28) = 1.28, *p* = 0.267, $\eta_{p}^{2}$ = 0.04, and *F* (1,28) = 3.07 *p* = 0.091, $\eta_{p}^{2}$ = 0.09, respectively].

## 4. Discussion

In the present study, we combined the Posner cueing task and the visual crowding task to investigate the temporal dynamics of endogenous and exogenous cues in alleviating visual crowding. The predictive validity of the cue was set to 75%. The behavioral results revealed that both endogenous and exogenous cues alleviated visual crowding but with differing temporal dynamics. Specifically, endogenous cues only relieved visual crowding at long SOAs, whereas exogenous cues worked at both short and long SOAs. At the electrocortical level, our analysis revealed that the suppression of N1 amplitudes caused by visual crowding was significantly alleviated by valid exogenous cues at short SOAs. In contrast, valid endogenous cues only alleviated this suppression at long SOAs. Moreover, valid exogenous cues induced smaller P3 amplitudes at a short SOA compared to invalid cues, whereas no such distinction was observed for endogenous cues.

In previous research, only two studies have contrasted the effects of endogenous and exogenous attention on crowding [9,27]. Our behavioral data, consistent with the findings of Gong et al. [9], demonstrated distinct effects of endogenous and exogenous attention on visual crowding, specifically in terms of the degree of reduction and differences in time course. On the one hand, compared to endogenous cues, exogenous cues had a greater alleviating effect on visual crowding. On the other hand, exogenous cues alleviated visual crowding at both long and short SOAs, whereas endogenous cues worked only at long SOAs. This result suggests that endogenous attention requires more time to alleviate visual crowding compared to exogenous attention.

In addition to behavioral evidence, our ERP data provided further support for the discrepancy in the time course of endogenous and exogenous attention in reducing crowding. The exogenous cue effectively reduced the suppression effect of crowding on the N1 component in the short-SOA condition. In contrast, the endogenous cue did not demonstrate this effect in the short-SOA condition, only demonstrating its effect in the long-SOA condition. This finding aligns with earlier studies suggesting that endogenous attention requires a much longer time for deployment compared to exogenous attention (e.g., [16,34,35]). More importantly, this finding suggests that the effects of exogenous and endogenous attention on crowding diverge before 200 ms during perceptual processing.

Previous studies have demonstrated that the N1 component is suppressed under crowding conditions compared to uncrowded conditions [28,30]. The findings of the current study revealed that the N1 component evoked by valid cues, whether endogenous or exogenous, exhibited a more negative amplitude compared to invalid cues. This finding indicates that both valid exogenous cues and valid endogenous cues can alleviate the effects of visual crowding compared to invalid ones. The visual N1 component reflects a discrimination process within the focus of attention [39], with stimuli presented at the attended locations being accompanied by a larger N1 compared to those presented at unattended locations [40,41,42]. This suggests that both endogenous and exogenous attention reduce crowding by enhancing attentional selection towards the target.

For the P3 amplitude, our study found that at the short SOAs, the P3 amplitude was significantly larger in the invalid exogenous cue condition than that in the valid exogenous cue condition, while no significant difference was observed for endogenous cues. The amplitude of P3 increases with task difficulty, reflecting a greater demand for cognitive resources (e.g., [31]). It is well documented that exogenous attention can be effectively deployed around 100 ms after cue onset (e.g., [16]). Therefore, valid exogenous cues can enhance the allocation of attentional resources toward the target even at short SOAs, which facilitates target discrimination. This was reflected by larger P3 amplitudes for valid exogenous cues than for invalid cues. Conversely, it is difficult for endogenous attention to exert its effect in a short SOA, and thus, no difference in P3 amplitude was found between valid and invalid cues. The present research also showed that at long SOAs, the P3 amplitude elicited by valid and invalid cues had no significant difference, neither for exogenous cues nor endogenous cues. This can be due to the potential that participants had sufficient time to allocate attention, even when the cue was invalid. Therefore, valid and invalid cues did not elicit significant changes in P3 amplitude at long SOAs.

It is worth noting that at long SOAs, valid endogenous cues reduced crowding compared to invalid endogenous cues. However, this distinction was reflected by N1 but not P3. We believe this result is relevant to the characteristics of endogenous attention. Endogenous attention is a voluntary system driven by goals [16,34,43]; thus, participants can flexibly determine whether to follow the cue according to the probability of valid trials [43,44,45]. Given the 75% predictive validity of the cue used in this study, participants tend to follow the cue. This implies that, for valid endogenous cues, participants still need to allocate substantial cognitive resources to shift their attention according to the cues. This could explain why there is no reduction in P3 under valid cues compared to invalid cues. Nevertheless, visual crowding is still reduced. This finding suggests that while both exogenous cues and endogenous attention can alleviate crowding, they involve different mechanisms.

Combining the behavioral and ERP results, the present study demonstrated that both exogenous and endogenous attention can alleviate visual crowding. This result aligns with the attentional resolution model of crowding. According to this model, the presence of flankers reduces the spatial resolution of visual attention, which creates a bottleneck in higher visual areas and leads to the emergence of crowding [10]. Both exogenous and endogenous attention cues enhance the attentional resolution at the cued location [16,21,34]; thus, they should reduce crowding, according to the attentional resolution model. The current study’s findings support this notion. The behavioral and ERP results also replicated the conclusion that there are differences in the time course of these two types of attention (Bowen et al. [27]; Gong et al. [9]), with the exogenous cue exerting its effects earlier than the endogenous cue. This finding is in accordance with previous research on the temporal dynamics of attention (e.g., [14,16,46]).

The findings of this study may hold important implications. Firstly, given the rapid effect of exogenous attention in alleviating visual crowding, in the design of visual displays, exogenous attention could be utilized to guide visual attention. For example, color contrast or spatial separation could be used to more effectively guide users’ attention, enabling key information to be quickly located and recognized even in complex visual environments. Secondly, in clinical contexts, these findings can be applied to improve the visual performance of particular populations. For instance, individuals with amblyopia are significantly affected by crowding (e.g., [47]). Unlike those with normal vision, they are significantly affected by visual crowding even in their central visual field. It may be possible to reduce visual crowding for individuals with amblyopia through attention training that enhances their endogenous attention capabilities, thereby assisting them in more effectively identifying target objects in complex visual environments.

However, there are some limitations to our study. Firstly, there was no baseline condition as a control, such as a neutral or no-cue condition, which makes it challenging to assess the absolute effects of both exogenous and endogenous cues compared to a baseline. The present experiment comprised 960 trials. Including neutral or no-cue trials would have raised the total number of trials to 1440, which could have been excessive and could have potentially led to fatigue effects. To prevent this, and also considering that the main purpose of this study was to compare the differences between exogenous and endogenous attention in alleviating crowding in target discrimination, we did not include neutral or no-cue conditions in our design. Given that the current study serves as a follow-up adopting a similar design to our previous study [9], both attentional cues are anticipated to alleviate crowding. Nonetheless, to definitively validate this hypothesis, further research incorporating a neutral or no-cue condition is necessary. Secondly, the exogenous cues typically reach their peak effects about 120 ms after the onset of the cues, while endogenous attention usually requires at least 300 ms for deployment (e.g., [34,35]). Therefore, in long-SOA conditions, exogenous attention may shift towards endogenous attention, thereby influencing the experimental results. Future studies may use eye-tracking technology to eliminate this confounding factor. Thirdly, the analysis of other components, N2pc in particular, could have provided a more comprehensive understanding of the attentional effects of exogenous and endogenous cues. N2pc is associated with selective attention and can be elicited by both endogenous and exogenous attention (e.g., [48]). The effects of these two cues on crowding may differ. Unfortunately, we did not mark the left and right visual fields separately and were unable to analyze the N2pc. This aspect is worthy of further investigation.

## 5. Conclusions

The present study provides behavioral and ERP evidence for the notion that both endogenous and exogenous attention can alleviate visual crowding. However, the results from the N1 component indicate that exogenous attention can exert its effects on visual crowding within 200 ms after cue onset, whereas endogenous attention necessitates a considerably longer period to alleviate visual crowding. Furthermore, as revealed by the P3 component, although both types of attention can reduce crowding, the effect of exogenous attention involves reducing cognitive resource investment. In contrast, the effect of endogenous attention does not include this aspect.

## Figures and Tables

**Table 1:** Reaction times (ms) for different SOAs and cue validity under endogenous and exogenous cue conditions.

Variable	Endogenous Cue		Exogenous Cue	
	Short SOA	Long SOA	Short SOA	Long SOA
Invalid cue	810.64 (166.16)	795.68 (144.00)	844.10 (180.29)	840.72 (200.00)
Valid cue	764.52 (137.17) **	745.35 (140.55) ***	755.41 (167.26) ***	750.32 (176.22) ***

Note. The asterisk indicates the significance relative to the invalid cue condition. ** *p* < 0.01; *** *p* < 0.001.

## Data Availability

The original data presented in this study are openly available in the Open Science Framework at https://osf.io/rv9fu/?view_only=b17e0d3888f94c4c802a17ed21a01c67.

## References

[1] Levi D.M. (2008). Crowding—An essential bottleneck for object recognition: A mini-review. Vis. Res..

[2] Whitney D., Levi D.M. (2011). Visual crowding: A fundamental limit on conscious perception and object recognition. Trends Cogn. Sci..

[3] Livne T., Sagi D. (2007). Configuration influence on crowding. J. Vis..

[4] Pelli D.G., Palomares M., Majaj N.J. (2004). Crowding is unlike ordinary masking: Distinguishing feature integration from detection. J. Vis..

[5] Põder E. (2008). Crowding with detection and coarse discrimination of simple visual features. J. Vis..

[6] Levi D.M., Carney T. (2009). Crowding in peripheral vision: Why bigger is better. Curr. Biol..

[7] Felisberti F.M., Solomon J.A., Morgan M.J. (2005). The role of target salience in crowding. Perception.

[8] Põder E. (2007). Effect of colour pop-out on the recognition of letters in crowding conditions. Psychol. Res..

[9] Gong M., Liu T., Liu X., Huangfu B., Geng F. (2023). Attention relieves visual crowding: Dissociable effects of peripheral and central cues. J. Vis..

[10] He S., Cavanagh P., Intriligator J. (1996). Attentional resolution and the locus of visual awareness. Nature.

[11] Scolari M., Kohnen A., Barton B., Awh E. (2007). Spatial attention, preview, and popout: Which factors influence critical spacing in crowded displays?. J. Vis..

[12] Scolari M., Awh E. (2019). Object-based biased competition during covert spatial orienting. Atten. Percept. Psychophys..

[13] Yeshurun Y., Rashal E. (2010). Precueing attention to the target location diminishes crowding and reduces the critical distance. J. Vis..

[14] Posner M.I. (1980). Orienting of attention. Q. J. Exp. Psychol..

[15] Dugué L., Merriam E.P., Heeger D.J., Carrasco M. (2020). Differential impact of endogenous and exogenous attention on activity in human visual cortex. Sci. Rep..

[16] Carrasco M. (2011). Visual attention: The past 25 years. Vis. Res..

[17] Jonides J., Long J.B., Baddeley A.D. (1981). Voluntary vs. automatic control over the mind’s eye’s. Attention and Performance IX.

[18] Giordano A.M., McElree B., Carrasco M. (2009). On the automaticity and flexibility of covert attention: A speed-accuracy trade-off analysis. J. Vis..

[19] Hein E., Rolke B., Ulrich R. (2006). Visual attention and temporal discrimination: Differential effects of automatic and voluntary cueing. Vis. Cogn..

[20] Liu T., Stevens S.T., Carrasco M. (2007). Comparing the time course and efficacy of spatial and feature-based attention. Vis. Res..

[21] Anton-Erxleben K., Carrasco M. (2013). Attentional enhancement of spatial resolution: Linking behavioural and neurophysiological evidence. Nat. Rev. Neurosci..

[22] Dakin S.C., Bex P.J., Cass J.R., Watt R.J. (2009). Dissociable effects of attention and crowding on orientation averaging. J. Vis..

[23] Freeman J., Pelli D.G. (2007). An escape from crowding. J. Vis..

[24] Kewan-Khalayly B., Migó M., Yashar A. (2022). Transient attention equally reduces visual crowding in radial and tangential axes. J. Vis..

[25] Tkacz-Domb S., Yeshurun Y. (2017). Spatial attention alleviates temporal crowding, but neither temporal nor spatial uncertainty are necessary for the emergence of temporal crowding. J. Vis..

[26] Montaser-Kouhsari L., Rajimehr R. (2005). Subliminal attentional modulation in crowding condition. Vis. Res..

[27] Bowen J.D., Alforque C.V., Silver M.A. (2023). Effects of involuntary and voluntary attention on critical spacing of visual crowding. J. Vis..

[28] Chicherov V., Plomp G., Herzog M.H. (2014). Neural correlates of visual crowding. Neuroimage.

[29] Peng C., Hu C., Chen Y. (2018). The temporal dynamic relationship between attention and crowding: Electrophysiological evidence from an event-related potential study. Front. Neurosci..

[30] Ronconi L., Bertoni S., Marotti R.B. (2016). The neural origins of visual crowding as revealed by event-related potentials and oscillatory dynamics. Cortex.

[31] Isreal J.B., Chesney G.L., Wickens C.D., Donchin E. (1980). P300 and tracking difficulty: Evidence for multiple resources in dual-task performance. Psychophysiology.

[32] Kok A. (2001). On the utility of P3 amplitude as a measure of processing capacity. Psychophysiology.

[33] Vila-Chã C., Vaz C., Oliveira A.S. (2022). Electrocortical activity in older adults is more influenced by cognitive task complexity than concurrent walking. Front. Aging Neurosci..

[34] Carrasco M., Barbot A. (2014). How attention affects spatial resolution. Cold Spring Harbor Symposia on Quantitative Biology.

[35] Cheal M., Lyon D.R. (1991). Central and peripheral precuing of forced-choice discrimination. Q. J. Exp. Psychol..

[36] Delorme A., Makeig S. (2004). EEGLAB: An open source toolbox for analysis of single-trial EEG dynamics including independent component analysis. J. Neurosci. Methods.

[37] Kim H., Luo J., Chu S., Cannard C., Hoffmann S., Miyakoshi M. (2023). ICA’s bug: How ghost ICs emerge from effective rank deficiency caused by EEG electrode interpolation and incorrect re-referencing. Front. Signal Process..

[38] Tian Y., Klein R.M., Satel J., Xu P., Yao D. (2011). Electrophysiological explorations of the cause and effect of inhibition of return in a cue–target paradigm. Brain Topogr..

[39] Vogel E.K., Luck S.J. (2000). The visual N1 component as an index of a discrimination process. Psychophysiology.

[40] Luck S.J., Hillyard S.A. (1994). Spatial filtering during visual search: Evidence from human electrophysiology. J. Exp. Psychol. Hum. Percept. Perform..

[41] Luck S.J., Hillyard S.A., Mouloua M., Woldorff M.G., Clark V.P., Hawkins H.L. (1994). Effects of spatial cuing on luminance detectability: Psychophysical and electrophysiological evidence for early selection. J. Exp. Psychol. Hum. Percept. Perform..

[42] Mangun G.R., Hillyard S.A. (1991). Modulations of sensory-evoked brain potentials indicate changes in perceptual processing during visual-spatial priming. J. Exp. Psychol. Hum. Percept. Perform..

[43] Müller H.J., Rabbitt P.M. (1989). Reflexive and voluntary orienting of visual attention: Time course of activation and resistance to interruption. J. Exp. Psychol. Hum. Percept. Perform..

[44] Kinchla R. (2014). The measurement of attention. Attention and Performance VIII.

[45] Sperling G., Melchner M.J. (1978). The attention operating characteristic: Examples from visual search. Science.

[46] Busse L., Katzner S., Treue S. (2008). Temporal dynamics of neuronal modulation during exogenous and endogenous shifts of visual attention in macaque area MT. Proc. Natl. Acad. Sci. USA.

[47] Hussain Z., Webb B.S., Astle A.T., McGraw P.V. (2012). Perceptual learning reduces crowding in amblyopia and in the normal periphery. J. Neurosci..

[48] Hickey C., Van Zoest W., Theeuwes J. (2010). The time course of exogenous and endogenous control of covert attention. Exp. Brain Res..

